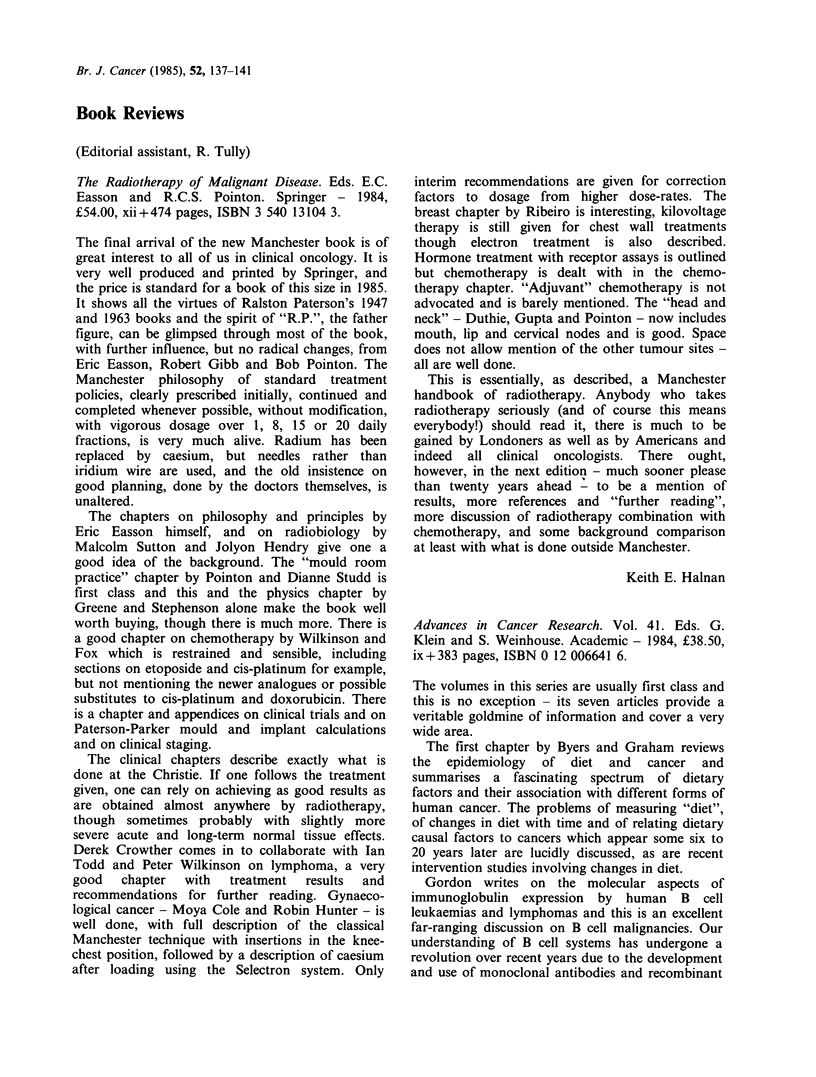# The Radiotherapy of Malignant Disease

**Published:** 1985-07

**Authors:** Keith E. Halnan


Br. J. Cancer (1985), 52, 137–141

### Book Reviews

(Editorial assistant, R. Tully)

*The Radiotherapy of Malignant Disease*. Eds. E.C. Easson and R.C.S. Pointon. Springer – 1984, £54.00, xii + 474 pages, ISBN 3 540 13104 3.

The final arrival of the new Manchester book is of great interest to all of us in clinical oncology. It is very well produced and printed by Springer, and the price is standard for a book of this size in 1985. It shows all the virtues of Ralston Paterson's 1947 and 1963 books and the spirit of "R.P.", the father figure, can be glimpsed through most of the book, with further influence, but no radical changes, from Eric Easson, Robert Gibb and Bob Pointon. The Manchester philosophy of standard treatment policies, clearly prescribed initially, continued and completed whenever possible, without modification, with vigorous dosage over 1, 8, 15 or 20 daily fractions, is very much alive. Radium has been replaced by caesium, but needles rather than iridium wire are used, and the old insistence on good planning, done by the doctors themselves, is unaltered.

The chapters on philosophy and principles by Eric Easson himself, and on radiobiology by Malcolm Sutton and Jolyon Hendry give one a good idea of the background. The "mould room practice" chapter by Pointon and Dianne Studd is first class and this and the physics chapter by Greene and Stephenson alone make the book well worth buying, though there is much more. There is a good chapter on chemotherapy by Wilkinson and Fox which is restrained and sensible, including sections on etoposide and cis-platinum for example, but not mentioning the newer analogues or possible substitutes to cis-platinum and doxorubicin. There is a chapter and appendices on clinical trials and on Paterson-Parker mould and implant calculations and on clinical staging.

The clinical chapters describe exactly what is done at the Christie. If one follows the treatment given, one can rely on achieving as good results as are obtained almost anywhere by radiotherapy, though sometimes probably with slightly more severe acute and long-term normal tissue effects. Derek Crowther comes in to collaborate with Ian Todd and Peter Wilkinson on lymphoma, a very good chapter with treatment results and recommendations for further reading. Gynaecological cancer – Moya Cole and Robin Hunter – is well done, with full description of the classical Manchester technique with insertions in the knee-chest position, followed by a description of caesium after loading using the Selectron system. Only interim recommendations are given for correction factors to dosage from higher dose-rates. The breast chapter by Ribeiro is interesting, kilovoltage therapy is still given for chest wall treatments though electron treatment is also described. Hormone treatment with receptor assays is outlined but chemotherapy is dealt with in the chemotherapy chapter. "Adjuvant" chemotherapy is not advocated and is barely mentioned. The "head and neck" – Duthie, Gupta and Pointon – now includes mouth, lip and cervical nodes and is good. Space does not allow mention of the other tumour sites – all are well done.

This is essentially, as described, a Manchester handbook of radiotherapy. Anybody who takes radiotherapy seriously (and of course this means everybody!) should read it, there is much to be gained by Londoners as well as by Americans and indeed all clinical oncologists. There ought, however, in the next edition – much sooner please than twenty years ahead – to be a mention of results, more references and "further reading", more discussion of radiotherapy combination with chemotherapy, and some background comparison at least with what is done outside Manchester.

Keith E. Halnan